# Patent and Exclusivity Status of Essential Medicines for Non-Communicable Disease

**DOI:** 10.1371/journal.pone.0051022

**Published:** 2012-11-30

**Authors:** Tim K. Mackey, Bryan A. Liang

**Affiliations:** 1 Institute of Health Law Studies, California Western School of Law, San Diego, California, United States of America; 2 Joint Doctoral Program on Global Health, University of California San Diego-San Diego State University, San Diego, California, United States of America; 3 San Diego Center for Patient Safety, University of California San Diego School of Medicine, San Diego, California, United States of America; 4 Department of Anesthesiology, University of California San Diego School of Medicine, San Diego, California, United States of America; Universidad Peruana Cayetano Heredia, Peru

## Abstract

**Objective:**

The threat of non-communicable diseases (“NCDs”) is increasingly becoming a global health crisis and are pervasive in high, middle, and low-income populations resulting in an estimated 36 million deaths per year. There is a need to assess intellectual property rights (“IPRs”) that may impede generic production and availability and affordability to essential NCD medicines.

**Methods:**

Using the data sources listed below, the study design systematically eliminated NCD drugs that had no patent/exclusivity provisions on API, dosage, or administration route. The first step identified essential medicines that treat certain high disease burden NCDs. A second step examined the patent and exclusivity status of active ingredient, dosage and listed route of administration using exclusion criteria outlined in this study.

**Materials:**

We examined the patent and exclusivity status of medicines listed in the World Health Organization’s (“WHO”) Model List of Essential Drugs (Medicines) (“MLEM”) and other WHO sources for drugs treating certain NCDs. i.e., cardiovascular and respiratory disease, cancers, and diabetes. We utilized the USA Food and Drug Administration Orange Book and the USA Patent and Trademark Office databases as references given the predominant number of medicines registered in the USA.

**Results:**

Of the 359 MLEM medicines identified, 22% (79/359) address targeted NCDs. Of these 79, only eight required in-depth patent or exclusivity assessment. Upon further review, no NCD MLEM medicines had study patent or exclusivity protection for reviewed criteria.

**Conclusions:**

We find that ensuring availability and affordability of potential generic formulations of NCD MLEM medicines appears to be more complex than the presence of IPRs with API, dosage, or administration patent or exclusivity protection. Hence, more sophisticated analysis of NCD barriers to generic availability and affordability should be conducted in order to ensure equitable access to global populations for these essential medicines.

## Introduction

Almost two-thirds of the 57 million global deaths occurring in 2008 were due to non-communicable diseases (“NCDs”), primarily: cardiovascular disease, diabetes, cancer and chronic respiratory disease, which are now the leading causes of deaths globally. [1], [2] In fact, almost 80% of NCD-related deaths occurs in low-and middle-income countries (“LMICs”), representing the most frequent causes of mortality on most continents, with the exception of Africa. [1], [3] Around the world, annual NCD mortality is projected to continue to rise, especially in LMICs, which are expected to experience the greatest increase in prevalence. [1], [4] Indeed, ∼80% of cardiovascular and diabetes mortality, nearly 90% of mortality from chronic obstructive pulmonary disease, and greater than two-thirds of cancer mortality occur in LMICs, though in low-income countries infectious diseases remain the major causes of mortality. [1], [2], [5] This rise in NCD prevalence, coupled with high out-of-pocket expenditures for medicines in LMICs, highlights a growing and disproportionate impact in resource-poor populations and the need to examine potential barriers to medication access. [1], [3], [6], [7].

With this epidemiological shift from communicable to NCDs [1], [4], [8], the international community is paying greater attention to this global health threat, with the UN General Assembly convened a high-level meeting in September 2011 with participating Member States on the prevention and control of NCDs. [9] While prevention is a predominant public health strategy [1], [10], availability and affordability to life-saving medications is also critical. This includes assessment of availability of generic essential medicines, whose presence generally results in greater availability of cheaper medications in comparison to innovator products that are often subject to IPRs. [6], [8] Indeed, studies have reported that LMICs can experience over 300% in pricing differentials between originator and lowest-price generics in the private sector. [7] In the case of HIV/AIDS, patent protection and related delayed market production of generic antiretroviral therapy (“ARVs”) led to pressure from civil society, eventual generic production and price negotiation, and the subsequent dramatic lowering of the cost of ARVs for resource-poor settings. [11].

Yet, comprehensive patent and exclusivity analysis on essential NCD medicines has not been adequately assessed. Previous studies have identified drugs on the World Health Organization’s (“WHO”) Model List of Essential Drugs (Medicines) (“MLEM”) have a low prevalence of patent exclusivity. [12] MLEM medicines are those satisfying priority health care needs and are selected on the basis of disease prevalence, safety, efficacy, and comparative cost-effectiveness. [13], [14] Yet, conversely, other studies have reported that certain essential medicines used to treat chronic diseases in LMICs may have limited availability and affordability, especially in public sector settings.[6]–[8] Specifically, studies have identified essential medicines treating diabetes, asthma, cardiovascular disease, and cancer as lacking availability and affordability.[15]–[18].

Hence, given rising prevalence of NCDs and urgent need for equitable access, we examined the state of patent and/or exclusivity for identified MLEM NCD medicines for supply-side factors associated with generic production and subsequent availability and affordability. This included determining if identified MLEM NCD medicines had patent or exclusivity protections that could preclude use of active pharmaceutical ingredient (API), MLEM-indicated formulation, or MLEM-indicated dosage by generic manufacturers.

## Methods

### 1. Overview

In general, we employed a two-phase approach to identify then analyze current MLEM NCD medications. In Phase I we began by identifying MLEM medicines treating diabetes, cancers, cardiovascular diseases, and respiratory diseases (“Targeted NCD Medicines”). These NCDs are major contributors to global disease burden and mortality. [1], [2], [6], [8], [19] In Phase II, we then examined the patent and exclusivity status of Targeted NCD Medicines using the FDA Approved Medicine Products with Therapeutic Equivalence Evaluation (“Orange Book”) and U.S. Patent and Trademark Office (“USPTO”) databases. The USA system was chosen for patent/exclusivity analysis as it is the largest pharmaceutical market globally, has easily accessible drug exclusivity and patent data sources (e.g., Orange Book), and is likely to have the greatest level of drug IPR protections. [20].

### 2. Phase I: Identifying Targeted NCD Medicines on MLEM

Specifically, to identify Targeted NCD Medicines, we used WHO data sources, i.e., current MLEM (17^th^ list) and WHO Essential Medicines Library. Currently, MLEM includes over 350 medicines and is updated every 2 years using evidence-based decision processes. [13] The MLEM is divided into two separate categories including “core” list medicines representing the minimum medicine needs for basic health systems, and a “complementary” list of essential medicines that may be less cost-effective or require diagnostic or monitoring facilities. [13], [14] Since 2002, cost-effectiveness comparisons are conducted but cost or affordability is no longer a reason for MLEM exclusion, nor is patent status of a medicine. [14] The MLEM, although not comprehensive, serves as an initial model list for countries in developing their own national formularies or policy. [14], [21].

Targeted NCD Medicines were identified by reviewing the most recently revised MLEM and searching for therapeutic subclasses (e.g., “cardiovascular medicines”) to determine if a drug addresses a targeted NCD. After identification of relevant subclass of drugs from MLEM, we used the WHO Essential Medicines Library to review all identified medicines for the “disease indication” to assess if the medicine was specifically indicated for a targeted NCD (note “disease indication” is not listed on the MLEM). Based on these results, a “Final NCD List” (see **Table 1**) was compiled for further review of associated patent/exclusivity data.

**Table 1:** Final Targeted NCD Medicines List.

INN/Compound	Category
Epinephrine	Cardiovascular disease, Respiratory disease
Allopurinol	Cancers
Asparaginase	Cancers
Bleomycin	Cancers
Calcium Folinate	Cancers
Carboplatin	Cancers
Chlorambucil	Cancers
Cyclophosphamide	Cancers
Cytarabine	Cancers
Dacarbazine	Cancers
Dactinomycin	Cancers
Daunorubicin	Cancers
Docetaxel	Cancers
Doxorubicin	Cancers
Etoposide	Cancers
Fluorouracil	Cancers
Hydroxycarbamide	Cancers
Ifosfamide	Cancers
Mercaptopurine	Cancers
Mesna	Cancers
Methotrexate	Cancers
Paclitaxel	Cancers
Procarbazine	Cancers
Thioguanine	Cancers
Vinblastine	Cancers
Vincristine	Cancers
Dexamethasone	Cancers
Hydrocortisone	Cancers
Methylprednisolone	Cancers
Prednisolone	Cancers
Tamoxifen	Cancers
Amitriptyline	Diabetes
Morphine	Cancers, Cardiovascular disease
Ondansetron	Cancers
Heparin Sodium	Cardiovascular disease
Protamine Sulfate	Cardiovascular disease
Tranexamic acid	Cardiovascular disease
Warfarin	Cardiovascular disease
Dextran 70	Cardiovascular disease
Factor IX Complex Concentrate	Cardiovascular disease
Factor VIII Concentrate	Cardiovascular disease
Human normal immunoglobulin	Cancers
Bisoprolol	Cardiovascular disease
Glyceryl trinitrate	Cardiovascular disease
Isosorbide dinitrate	Cardiovascular disease
Verapamil	Cardiovascular disease
Amiodarone	Cardiovascular disease
Digoxin	Cardiovascular disease
Lidocaine	Cardiovascular disease
Amlodipine	Cardiovascular disease
Enalapril	Cardiovascular disease
Hydralazine	Cardiovascular disease
Hydrochlorothiazide	Cardiovascular disease, Diabetes
Methyldopa	Cardiovascular disease
Sodium Nitroprusside	Cardiovascular disease
Dopamine	Cardiovascular disease
Furosemide	Cardiovascular disease, Diabetes
Acetylsalicylic Acid	Cardiovascular disease
Streptokinase	Cardiovascular disease
Simvastatin	Cardiovascular disease
Amiloride	Cardiovascular disease
Mannitol	Cardiovascular disease
Spironolactone	Cardiovascular disease
Metoclopramide	Cancers
Glibenclamide	Diabetes
Glucagon	Diabetes
Insulin Injection (soluble)	Diabetes
Immediate-acting insulin	Diabetes
Metformin	Diabetes
Intraperitoneal Dialysis Solution	Diabetes
Nicotine replacement therapy	Cardiovascular disease, Respiratory disease
Beclometasone	Respiratory disease
Budesonide	Respiratory disease
Ipratropium bromide	Respiratory disease
Salbutamol	Respiratory disease
Glucose	Diabetes
Xylometazoline	Respiratory disease
Prostaglandin E1	Cardiovascular disease
Surfactant	Respiratory disease

Sources: WHO Model List of Essential Medicines, 17^th^ List.

### 3. Phase II: Assessing Patent/Exclusivity Status of Targeted NCD Medicines

Once the Final NCD List was compiled, we then analyzed the patent/exclusivity status of these medicines using the FDA Orange Book, which lists all medicine products approved under a New Drug Application (NDAs) (for innovator products) or an Abbreviated New Medicine Application (ANDAs) (for generic products). Patent/exclusivity disclosure contained in the Orange Book are made part of applications submitted by manufacturers to FDA during the NDA or ANDA process. [22].

FDA-granted exclusive data and marketing rights are also reported in the Orange Book. Exclusivity differs from patent protection in that it provides statutory exclusion of others from marketing or use of originator’s test data for subsequent drug applications. Exclusivity terms can run concurrently or in seriatim. [23] USA exclusivity includes data exclusivity for New Chemical Entities or new active moiety (5-years), and marketing exclusivity for Orphan Drug Exclusivity (“ODE”) (7-years), Pediatric Exclusivity (6-months), Patent Challenge Exclusivity (180-days), and changes and submission of supplemental applications (e.g., new uses/indications for already approved products) (3-years). [23] We cross-referenced the API of identified NCD Targeted Medicines with the Orange Book for any patents/exclusivity designation associated with the medication. We then reviewed the relevant drug authorizations for patents/exclusivity designation in MLEM-indicated: (a) API; (b) dosage; and (c) route of administration.

Targeted NCD Medicines were not included as being protected by patent/exclusivity terms if they: (i) had different administration method from that in the MLEM; (ii) were not listed as a reference listed drug; (iii) were classified as having therapeutic equivalence; or (iv) did not make a drug substance claim (“Orange Book Exclusion Criteria”). We did not include drugs with these characteristics because not having the same MLEM-listed administration indicates a different method of administration or formulation than that recommended under the MLEM; a drug not listed as a reference listed drug indicates it is generally not an innovator product (i.e., it is a generic or other formulation); therapeutic equivalence designation indicates these drugs are pharmaceutical equivalents (contain the same active ingredient(s); dosage form and route of administration; and strength, again, a generic); and not making a drug substance claim indicates that the drug does not make a claim on the API. [22].

For Targeted NCD Medicines with drug applications indicating patent/exclusivity protection not meeting the above Orange Book Exclusion Criteria, we used the USPTO comprehensive database of USA-filed patents through its Patent Application Information (“PAIR”) system to specifically examine applicable patent claims to assess if they were associated with generic production of the API, MLEM indicated route of administration, and MLEM dosage. Here, we did not include patents/exclusivity making claims for (a) an administration or indication *not* addressed in MLEM; (b) combination products of several drugs/active substances; (c) method patents to manufacturer/process drugs; (d) derivatives, formulations, or compositions containing active substances but not associated with resultant generic production of API; (e) limited exclusivity designation such as Orphan Drug status; and (f) patents for mechanical delivery devices (“USPTO Exclusion Criteria”). This step revalidated the Orange Book Exclusion Criteria and allowed us to look at patent/exclusivity designations that were unclear or that indicated possible protection per Orange Book data.

**Figure 1:**
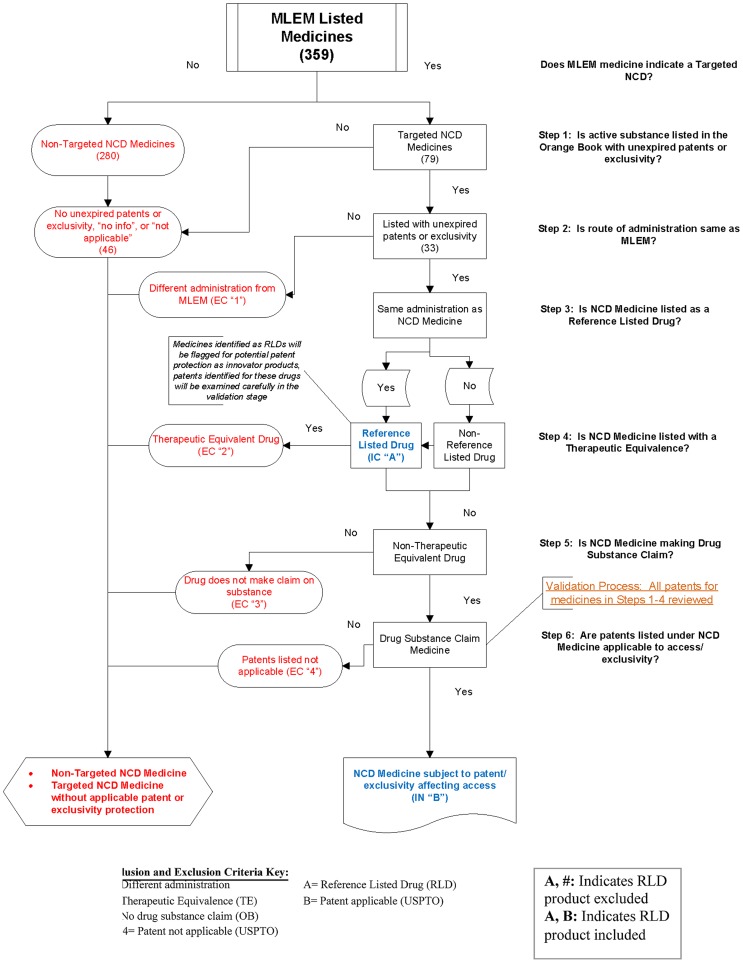
Patent Inclusion/Exclusion Decision Algorithm and Explanation.

Our decision algorithm for inclusion/exclusion criteria is provided in **Figure 1**. A summary of the Orange Book and USPTO Exclusion Criteria and examples of their application is provided in **Table 2**. Searches and analysis were conducted from June -August 2011.

**Table 2:** Orange Book and USPTO Non-Inclusion Criteria Summary and Examples.

Orange Book Non-Inclusion Criteria and Examples		
Exclusion Criteria	NCD Medicine	Reason
No patent/exclusivity info	Carboplatin	23 drug applications, none with unexpired patent/exclusivity
Different administration/dosage from MLEM	Fluorouracil	Carac (PN) identified but with route of administration as topical cream not covered in MLEM
Classified as Therapeutic Equivalence	Gilbernclamide (USAN: Glyburide)	Glucovance (PN) had patent/exclusivity data, but further examination determined TE classification and patent associated with combination uses
Did not make active drug substance claim	Isosorbide Dinitrate	Bidil (PN) listed as RLD, but remaining patents not associated with active drug substance
**USPTO Exclusion Criteria and Examples**		
Combination product of 1 active substance	Amlodipine	Cadut (PN) identified but combination therapy of amlodipine and atorvastatin
Method patent to manufacture/process drug	Cytarabine	Depocyt (PN) had patent/exclusivity data, but further examination determined method patent for treatment
Patents for other delivery devices	Nicotine Replacement Therapy	Nicorette and Committ (PN) identified with patent/exclusivity data, but further examination showed different delivery device then MLEM-indicated (e.g. trilaminate film and troche/lozenge)

## Results

### 1. Phase I MLEM NCD List Results

Phase I showed close to one-quarter, 22% (79/359) of all medicines on MLEM are used to treat a targeted NCD. Cancer and cardiovascular diseases make up the bulk of Targeted NCD Medicines addressed by MLEM (**Table 3**).

**Table 3:** Frequency of Therapeutic Category for Targeted NCD Medicines.

Category	Respiratory	Cardiovascular	Diabetes	Cancers
Targeted NCD Medicines (n=359)	2.23%	8.91%	2.23%	9.47%

**Additional Note:** For Targeted NCD medicines which addressed more than one Targeted NCD, frequency was attributed to all categories addressed.

### 2. Phase II Patent/Exclusivity Results

In Phase II, initial Orange Book patent searches on the 79 medicines in the Final NCD List showed only 42% (33) of these medicines had some type of positive results for patent and exclusivity protection, 43% (34) in negative results (i.e., no patent exclusivity of any type), 10% (8) which were not covered by the Orange Book (i.e., certain biologics, dialysis solutions, and insulin), and 5% (4) with no information available.

Using these results, we assessed all associated patent and exclusivity terms of the 33 Targeted NCD Medicines with positive results. Of these 33 identified Targeted NCD Medicines, only 8 had at least one patent/exclusivity term that did not meet the Orange Book Exclusion criteria.

These 8 remaining drugs then were assessed using patent/exclusivity data from USPTO data sources. These included calcium folinate (in USA: leucovorin), doxorubicin, amlodipine, hydrochlorothiazide, simvastatin, metformin, budesonide, and salbutamol (in USA: albuterol). Applying our USPTO Exclusion Criteria, we found that none of these drugs had patent claims that would impede generic manufacture of API, nor the MLEM-indicated route of administration or dosage (**Table 4**). Generic versions of drugs containing the API for all 8 of these drugs are commercially available.

**Table 4:** NCD Drugs and USPTO Criteria for Non-Inclusion in IPR Barrier Analysis.

NCD Drugs	USPTO Criteria for Non-Inclusion
calcium folinate, amlodipine,hydrochlorothiazide, simvastatin,and metformin	Excluded as a combination product not the MLEM active substance
doxorubicin	Exclusivity designated for Orphan Disease indication only
amlodipine	Identified patents were for formulation, process, dosage or methods

However, upon careful examination of patent claims for these 8 drugs, we found that two respiratory medications, salbutamol and budesonide, have patent/exclusivity data for MLEM-indicated formulation/administration via inhalers and their related administration technology that may impede generic availability. Yet, this IPR protection may be an artifact of USA prohibition of inhalers containing chlorofluorocarbons (“CFCs”), scheduled to be phased out of use due to negative environmental impact. [24] Due to the prohibition of CFC inhalers, the FDA has recommended use of hydrofluoroalkane (“HFA”) propellants. [24] Yet, no generic forms of HFA propellants are available for salbutamol, and, similarly, patents may also affect availability of HFA administration and delivery devices of budesonide. These findings illustrate the complexity and dynamic nature of IPR provisions that may impede generic production that can result in lack of availability or affordability of essential NCD medications.

## Discussion

We found that none of the Targeted NCD Medicines have applicable patent/exclusivity provisions that could hinder possible generic production of the API or MLEM-indicated formulation or dosage in our study. This implies, if these results are generalizable, that availability, affordability and delivery of essential NCD medicines, particularly in LMICs where studies have already identified access limitations, may be affected by other and/or additional considerations. With literally no WHO MLEM-based NCD drugs under our criteria being subject to API, dosage, or administration patent and/or exclusivity provisions, generic production and subsequent affordability of NCD drugs appears not to be solely an IPR issue. Hence, strategies to improve generic availability to NCD essential medicines must take into account other factors associated with medicines access that have been previously identified.[25]–[28].

Such strategies should include assessment using a comprehensive definition of “access” including concurrent definitions of physical availability, affordability, geographic accessibility and acceptability that affect both the supply and demand considerations, which are beyond the scope of this paper. [29], [30] While in this paper we limit our findings to and use of the term “access” to a narrow working definition of supply-side factors of availability and affordability influenced by generic production, indeed, several additional factors may limit greater NCD drug access, production and uptake. These include demand-side aspects of availability including prescribing practices, lack of generic substitution/procurement, acceptability of medicines for prescribers and users, greater public and private sector health financing, and better utilization of available medications.[6], [8], [18], [31]–[33].

In the scope of factors associated with IPRs, combination therapies that include multiple active substances may also be clinically efficacious, though are not covered under patent exclusivity for a single NCD Medicine active substance or the MLEM. This includes the suggested use of polypills (a combination drug with multiple API) for the prevention of cardiovascular disease that has been shown to be potentially clinically beneficial. [34] Though use of polypills may reduce cost and increase availability if API is off-patent, they may also be subject to patent protection for the formulation or may not otherwise be commercially attractive for manufacturers. [35] In addition, methods of administration (such as lozingers, aerosol formulations, pen injectors, etc.) that can have better uptake in certain patient populations may also be subject to patent protection and not covered in the MLEM.

Also, as in the case of calcium folinate and metformin, even if a medicine is no longer under patent protection, there may be lack of sufficient generic or off-patent brand name manufacturing capacity or incentives to meet clinical needs. In these cases, factors (such as low reimbursement as a result of private and public policies) may have an effect on production and investment in manufacturing and export for these essential drugs, leading to shortages. [36], [37] This is especially worrisome given studies reporting significantly lower availability of chronic disease medications in public sector settings and high-costs in the private sector for LMICs. [6], [8] Consequentially this creates a dual burden of limited availability at the public level (which may provide more affordability or free access), and lack of affordability at the private level for patients who are already predominantly paying out-of-pocket and are resource-constrained.

It should also be noted that the MLEM is *not* meant to be a comprehensive list of all medications necessary to address the plethora of debilitating conditions NCDs pose and their accompanying complex and ongoing treatment regimes. [7], [21] Indeed, outside of the MLEM, there may be other NCD drugs that are clinical efficacious but are not included on it. This includes a number of new medicines for the treatment of cancer that have been identified as being excluded from the MLEM. [38].

In addition, given both developing and developed country needs for a full suite of NCD drugs, the “patent cliff”, which represents dates when blockbuster drug patents/exclusivity for innovator drugs terminate, may present generic manufacturing opportunities and the chance to more proactively assess inclusion of therapies transitioning in cost-effectiveness for the MLEM. [39] Indeed, these pharmaceuticals that are coming off-patent will likely experience rapid, significant price reductions from generic production. [40] MLEM inclusion for these products could aid in providing technical assistance and expedite selection and inclusion on national drug formularies by alerting member states to the availability of lower-cost generic versions that would subsequently make these treatments more cost-effective and affordable.

To promote these potential benefits, WHO may wish to revisit the MLEM review process to more proactively assess if these NCD products coming off-patent adequately meet the criteria for safety, efficacy, and *increased* comparative cost-effectiveness outlined in the MLEM. Efforts should be made to actively engage generic manufacturers to address the need for these medications in underserved markets increasingly suffering from a growing burden of NCDs. These efforts could also extend to more active promotion of local manufacturing of essential NCD medicines to potentially increase economic benefits, availability, and achieve cost reduction. [6], [41], [42].

Further, potential expansion of the MLEM complimentary list to incorporate this proactive review of NCD medicines with expiring patent/exclusivity protection, may be a potential method to identify and encourage generic manufacturers and national drug formularies to develop, list, and invest in these products. These efforts would create a more dynamic MLEM, and potentially enable speedier access to needed medicines if they were appropriately deemed “essential.”

### Study Limitations

It is important to note that the WHO MLEM represents only a portion of medicines available for the effective treatment of NCDs, and other strategic medical approaches may be necessary for efficacious clinical treatment and therefore may not be included on this list. Further, national drug formularies and established standard of care provisions or clinical practice guidelines for medical professionals may differ from those medicines recommended in the MLEM.

In addition, a small subset of Targeted NCD Medicines on the MLEM were not assessed or did not have assessable information. The reasons for this includes: (a) medicine is a metabolite or intermediary; (b) biologic product which were not assessed for patentability/exclusivity; (c) general health product (such as insulin, human normal immunoglobulin) not covered under patent protection; or (d) substance/ingredient used in manufacturer/process of medicine, but not listed in Orange Book. However, these represented only 5% of all identified Targeted NCD Medicines.

In addition, we only assessed patent status of identified NCD Medicines using the FDA Orange Book, which limits the scope of our analysis to the USA. However, these results would tend to be overinclusive in IPRs due to securing rights in the globe’s largest drug market would generally be a priority for pharmaceutical manufacturers.

It should also be noted that this study methodology did not survey non-public databases for drug patent/exclusivity information and is limited to the USA. We also did not specifically assess the family patent status of NCD medicines. This includes determining from the family of patents the applicable base patent (the earliest patent for the API) and any API supplementary or ancillary patents. We limited our searches to Orange Book data and publicly accessible databases. However, if a base patent had remaining patent or exclusivity terms it should be disclosed in the Orange Book. If the base patent was expired, then it should not appear in these search results. For future study, detailed examination of the patent family for all Targeted NCD Medicines may provide further insights.

In addition, our data sources have certain limitations. It is possible patent rights registered and afforded in the Orange Book may not be recognized by other sovereignties. There may also be errors in the Orange book, e.g., API may or may not be registered or approved or may or may not be afforded additional patent protection. Hence, the population of drugs included in this study may reflect these errors as well, although the Orange Book is the standard source for USA-based IPR information. In addition, patent information reported in the Orange Book is based on NDA and ANDA applications and is not validated by the FDA. Though applicants must attest the information contained in an application is accurate, the FDA does not have a process to remove such information if it is inaccurate. Disputes can be filed with the FDA regarding inaccurate patent information, but it is ultimately the responsibility of the applicant to withdraw or amend patent information. Generally, the FDA relies on the patent litigation system to adjudicate these kinds of disputes.

### Concluding Remarks

It has been 8 years and four rounds of updates to the MLEM since Attaran’s pioneering study reported that only a few essential medicines are patented. The results from this study show that little has changed in relation to patents/exclusivity status for the specific subclasses of essential medicines needed to treat NCDs. This study provides important preliminary data on the association of patents/exclusivity for generic production, availability and affordability of NCD essential medicines; however, further research is necessary. These efforts should include examination of converging mutual benefits from strategies of IPR management, critical assessment of non IPR-factors that may impede availability and affordability, and possible reexamination of the process of review/update of the MLEM. [25] Finally, global health policy solutions should be directed towards a comprehensive menu of evidence-based interventions and improved global governance to improve access to medicines, while also identifying and adequately addressing the multitude of barriers to global health progress in the control of NCDs.

## References

[1] World Health Organization (2011) Global status report on noncommunicable diseases 2010. World Health Organization Website. Available: http://www.who.int/nmh/publications/ncd_report2010/en/. Accessed 1 November 2012.

[2] Alawan A (2011) WHO: “NCDs Now the Leading Cause of Deaths Globally” Foreign Affairs Website. Available:http://www.foreignaffairs.com/discussions/interviews/who-ncds-now-the-leading-cause-of-deaths-globally. Accessed 16 July 2012.

[3] Daar AS, Singer PA, Leah Persad D, Pramming SK, Matthews DR, et al (2007) Grand challenges in chronic non-communicable diseases. Nature 450: 494–496 doi:10.1038/450494a 18033288 10.1038/450494a

[4] Miranda JJ, Kinra S, Casas JP, Davey Smith G, Ebrahim S (2008) Non-communicable diseases in low- and middle-income countries: context, determinants and health policy. Trop Med Int Health 13: 1225–1234 doi:10.1111/j.1365–3156.2008.02116.x 18937743 10.1111/j.1365-3156.2008.02116.x PMC2687091

[5] World Health Organization (n.d.) The top 10 causes of death. World Health Organization Website. Available:http://www.who.int/mediacentre/factsheets/fs310/en/index.html. Accessed 30 July 2012.

[6] Mendis S, Fukino K, Cameron A, Laing R, Filipe A Jr, et al (2007) The availability and affordability of selected essential medicines for chronic diseases in six low-and middle-income countries. Bull World Health Organ 85: 279–288 doi:10.2471/BLT.06.033647 17546309 10.2471/BLT.06.033647 PMC2636320

[7] Cameron A, Ewen M, Rossdegnan D, Ball D, Laing R (2009) Medicine prices, availability, and affordability in 36 developing and middle-income countries: a secondary analysis. The Lancet 373: 240–249 doi:10.1016/S0140–6736(08)61762–6 10.1016/S0140-6736(08)61762-6 19042012

[8] Cameron A, Roubos I, Ewen M, Mantel-Teeuwisse AK, Leufkens HGM, et al (2011) Differences in the availability of medicines for chronic and acute conditions in the public and private sectors of developing countries. Bull World Health Organ 89: 412–421 doi:10.2471/BLT.10.084327 21673857 10.2471/BLT.10.084327 PMC3099556

[9] Mamudu HM, Yang JS, Novotny TE (2011) UN resolution on the prevention and control of non-communicable diseases: an opportunity for global action. Glob Public Health 6: 347–353 doi:10.1080/17441692.2011.574230 21607893 10.1080/17441692.2011.574230

[10] World Health Organization (2008) 2008–2013 Action Plan for the Global Strategy for the Prevention and Control of Noncommunicable Diseases. World Health Organization Website. Available:http://www.who.int/nmh/Actionplan-PC-NCD-2008.pdf. Accessed 16 July 2012.

[11] Hoen E', Berger J, Calmy A, Moon S (2011) Driving a decade of change: HIV/AIDS, patents and access to medicines for all. J Int AIDS Soc 14: 15 doi:10.1186/1758–2652–14–15 21439089 10.1186/1758-2652-14-15 PMC3078828

[12] Attaran A (2004) How do patents and economic policies affect access to essential medicines in developing countries? Health Aff (Millwood) 23: 155–166. 10.1377/hlthaff.23.3.155 15160813

[13] World Health Organization (2011) WHO Model List of Essential Medicines: 17th list. World Health Organization Website. Available:http://whqlibdoc.who.int/hq/2011/a95053_eng.pdf. Accessed 16 July 2012.

[14] Mirza Z (2008) Thirty years of essential medicines in primary health care. East Mediterr Health J 14 Suppl: S74–S81 19205606

[15] Gill GV, Yudkin JS, Keen H, Beran D (2011) The insulin dilemma in resource-limited countries. A way forward? Diabetologia 54: 19–24 doi:10.1007/s00125–010–1897–3 20835860 10.1007/s00125-010-1897-3

[16] Cruz AA, Bousquet PJ (2009) The unbearable cost of severe asthma in underprivileged populations. Allergy 64: 319–321 doi:10.1111/j.1398–9995.2009.02026.x 19284405 10.1111/j.1398-9995.2009.02026.x

[17] Kishore SP, Vedanthan R, Fuster V (2011) Promoting Global Cardiovascular Health. Journal of the American College of Cardiology 57: 1980–1987 doi:10.1016/j.jacc.2010.12.029 21565635 10.1016/j.jacc.2010.12.029

[18] Farmer P, Frenk J, Knaul FM, Shulman LN, Alleyne G, et al (2010) Expansion of cancer care and control in countries of low and middle income: a call to action. Lancet 376: 1186–1193 doi:10.1016/S0140–6736(10)61152-X 20709386 10.1016/S0140-6736(10)61152-X

[19] Mathers CD, Loncar D (2006) Projections of Global Mortality and Burden of Disease from 2002 to 2030. PLoS Med 3: e442 doi:10.1371/journal.pmed.0030442 17132052 10.1371/journal.pmed.0030442 PMC1664601

[20] IMS (2010) IMS Health Forecasts Global Pharmaceutical Market Growth of 5–7 Percent in 2011, Reaching $880 Billion. IMS Health Website. Available:http://www.imshealth.com/portal/site/ims/menuitem.d248e29c86589c9c30e81c033208c22a/?vgnextoid=119717f27128b210VgnVCM100000ed152ca2RCR. Accessed 16 July 2012.

[21] World Health Organization (2000) The Use of Essential Drugs. World Health Organization Website Available:http://whqlibdoc.who.int/trs/WHO_TRS_895.pdf. Accessed 16 July 2012.

[22] U.S. Food and Drug Administration (2011) Approved Drug Products with Therapeutic Equivalence Evaluations: 31st Edition. US Food and Drug Administration.

[23] U.S. Food and Drug Administratio (n.d.) Frequently Asked Questions on Patents and Exclusivity. FDA Website. Available:http://www.fda.gov/Drugs/DevelopmentApprovalProcess/ucm079031.htm. Accessed 16 July 2012.

[24] Metered Dose Inhalers: The Transition to Ozone-Safe Propellants (n.d.) Metered Dose Inhalers: The Transition to Ozone-Safe Propellants. US EPA Website. Available:http://www.epa.gov/ozone/title6/exemptions/inhalers.html. Accessed 16 July 2012.

[25] Mackey TK, Liang BA (2012) Promoting global health: utilizing WHO to integrate public health, innovation and intellectual property. Drug Discov Today. doi:10.1016/j.drudis.2012.06.012. 10.1016/j.drudis.2012.06.012 22728776

[26] Cohen JC, Illingworth P (2003) The dilemma of intellectual property rights for pharmaceuticals: the tension between ensuring access of the poor to medicines and committing to international agreements. Dev World Bioeth 3: 27–48. 14577451 10.1111/1471-8847.00058

[27] Beran D, McCabe A, Yudkin JS (2008) Access to medicines versus access to treatment: the case of type 1 diabetes. Bull World Health Organ 86: 648–649. 18797626 10.2471/BLT.07.048710 PMC2649458

[28] Abegunde D (2011) Background paper: Essential Medicines for Non-Communicable Diseases (NCDs). World Health Organization Website. Available:http://www.who.int/medicines/areas/policy/access_noncommunicable/EssentialMedicinesforNCDs.pdf. Accessed 16 July 2012.

[29] Defining and Measuring Access to Essential Drugs, Vaccines, and Health Commodities: Report of the WHO-MSH Consultative Meeting, Ferney-Voltaire, France, December 11–13, 2000 (2000) Ferney-Voltaire, France, December 11–13, 2000. Management Sciences for Health Website. Available:http://www.msh.org/seam/reports/Access_Meeting_Ferney_Voltaire_1.pdf. Accessed 22 October 2012.

[30] Peters DH, Garg A, Bloom G, Walker DG, Brieger WR, et al (2008) Poverty and access to health care in developing countries. Ann NY Acad Sci 1136: 161–171. 17954679 10.1196/annals.1425.011

[31] Chua GN, Hassali MA, Shafie AA, Awaisu A (2010) A survey exploring knowledge and perceptions of general practitioners towards the use of generic medicines in the northern state of Malaysia. Health Policy 95: 229–235 doi:10.1016/j.healthpol.2009.11.019 20044165 10.1016/j.healthpol.2009.11.019

[32] Patel A, Gauld R, Norris P, Rades T (2009) “This body does not want free medicines”: South African consumer perceptions of drug quality. Health Policy Plan 25: 61–69 doi:10.1093/heapol/czp039 19726560 10.1093/heapol/czp039

[33] Carrera PM (2009) Ethical Prescribing. Health Affairs 28: 1861–1862 doi:10.1377/hlthaff.28.6.1861-a 19887433 10.1377/hlthaff.28.6.1861-a

[34] Rodgers A, Patel A, Berwanger O, Bots M, Grimm R, et al (2011) An international randomised placebo-controlled trial of a four-component combination pill (“polypill”) in people with raised cardiovascular risk. PLoS ONE 6: e19857 doi:10.1371/journal.pone.0019857 21647425 10.1371/journal.pone.0019857 PMC3102053

[35] Guglietta A, Guerrero M (2008) Issues to consider in the pharmaceutical development of a cardiovascular polypill. Nature 6: 112–119 doi:10.1038/ncpcardio1424 10.1038/ncpcardio1424 19104518

[36] Steinbrook R (2009) Drug Shortages and Public Health. N Engl J Med. 361: 1525–1527 doi:10.1056/NEJMp0906922 10.1056/NEJMp0906922 19828529

[37] Connelly P, Quinn BP (2011) Manufacturing decline yields drug shortages. Science 333: 156–157 doi:10.1126/science.333.6039.156-b 10.1126/science.333.6039.156-b 21737721

[38] Miano P (2011) Approval, ownership, market structure, and placement on WHO EML for 100 new cancer NMEs on NCI alpha list. Knowledge Ecology International Website. Available:http://keionline.org/rn2011-1. Accessed 30 July 2012.

[39] The patent cliff steepens (2011) Nat Rev Drug Discov. 10: 12–13 doi:10.1038/nrd3356 10.1038/nrd3356 21193859

[40] Hollis A, Liang BA (2006) An Assessment of the Effect of Authorized Generics on Consumer Prices. Generic Pharmaceutical Association Website. Available:http://www.gphaonline.org/sites/default/files/GPhA_AG_Study.pdf. Accessed 16 July 2012.

[41] Pharmaceutical production/supply capacity (2002) Pharmaceutical production/supply capacity. UNCTAD Website. Available:http://www.unctad.org/templates/Page.asp?intItemID=4567&lang=1. Accessed 28 August 2011.

[42] Seiter A (2005) HNP Brief #3 Pharmaceuticals: Local Manufacturing. World Bank: 1–5. World Health Organization Website. Available: http://apps.who.int/medicinedocs/documents/s16758e/s16758e.pdf. Accessed 1 November 2012.

